# High Resolution Imaging of Temporal and Spatial Changes of Subcellular Ascorbate, Glutathione and H_2_O_2_ Distribution during *Botrytis cinerea* Infection in Arabidopsis

**DOI:** 10.1371/journal.pone.0065811

**Published:** 2013-06-05

**Authors:** Uwe K. Simon, Lisa M. Polanschütz, Barbara E. Koffler, Bernd Zechmann

**Affiliations:** Institute of Plant Sciences, University of Graz, Graz, Austria; Tsinghua University, China

## Abstract

In order to study the mechanisms behind the infection process of the necrotrophic fungus *Botrytis cinerea*, the subcellular distribution of hydrogen peroxide (H_2_O_2_) was monitored over a time frame of 96 h post inoculation (hpi) in *Arabidopsis thaliana* Col-0 leaves at the inoculation site (IS) and the area around the IS which was defined as area adjacent to the inoculation site (AIS). H_2_O_2_ accumulation was correlated with changes in the compartment-specific distribution of ascorbate and glutathione and chloroplast fine structure. This study revealed that the severe breakdown of the antioxidative system, indicated by a drop in ascorbate and glutathione contents at the IS at later stages of infection correlated with an accumulation of H_2_O_2_ in chloroplasts, mitochondria, cell walls, nuclei and the cytosol which resulted in the development of chlorosis and cell death, eventually visible as tissue necrosis. A steady increase of glutathione contents in most cell compartments within infected tissues (up to 600% in chloroplasts at 96 hpi) correlated with an accumulation of H_2_O_2_ in chloroplasts, mitochondria and cell walls at the AIS indicating that high glutathione levels could not prevent the accumulation of reactive oxygen species (ROS) which resulted in chlorosis. Summing up, this study reveals the intracellular sequence of events during *Botrytis cinerea* infection and shows that the breakdown of the antioxidative system correlated with the accumulation of H_2_O_2_ in the host cells. This resulted in the degeneration of the leaf indicated by severe changes in the number and ultrastructure of chloroplasts (e.g. decrease of chloroplast number, decrease of starch and thylakoid contents, increase of plastoglobuli size), chlorosis and necrosis of the leaves.

## Introduction

The infection of plants by necrotrophic pathogens is characterized by the release of phytotoxins, cell wall degrading enzymes and an oxidative burst which is triggered by the pathogen and finally leads to plant cell death. Early visible signs of these events are the appearance of chlorotic regions followed by necrosis [1]–[6]. The final stage of a necrotrophic infection process is a rapid decomposition of the plant material and its conversion into fungal biomass [6]. Antioxidants such as ascorbate and glutathione are counteracting the accumulation of ROS in order to keep the oxidative burst under control and limit it to the IS [5]. Nevertheless, during successful necrotrophic infections the antioxidative capacity of a plant gets eventually exhausted to an extent where the accumulation of ROS, especially H_2_O_2_, leads to severe ultrastructural damages which subsequently results in cell death [7]–[11]. Antioxidants such as ascorbate and glutathione do not only protect plants against oxidative stress but are also involved in sensing, signaling and activating plant defense [12]–[14]. Thus, the degree of H_2_O_2_ accumulation in correlation with the antioxidative capacity during infection is crucial for the survival of the plant. Even though the importance of H_2_O_2_ and antioxidants for the defense against necrotrophic fungal pathogens are well understood it still remains unclear in which cell compartments H_2_O_2_ accumulates during necrotrophic fungal infection and how it correlates to subcellular contents of antioxidants. Such information is crucial to understand the compartment-specific mechanisms behind the infection process of necrotrophic pathogens.

One of the best studied necrotrophic fungal pathogens is *Botrytis cinerea* [15]. During the early stages of *Botrytis cinerea* infection ROS accumulation has been observed around the penetrated cell walls, at the plasma membrane and inside cells close at and surrounding the necrotic IS [16]–[20]. In the later stages ROS accumulation advances inside the infected tissue, and results in the death of the infected organ [3], [11], [19], [20]. The successful establishment of *Botrytis cinerea* infection is due to the production of phytotoxins, cell wall degrading enzymes and the oxidative burst which eventually leads to the death of the cells [2], [3], [6]. Due to the accumulation of ROS a change in antioxidant levels is commonly observed. Generally, a decrease in the amount of ascorbate and glutathione has been observed in the later stages of the infection whereas during the early stages unchanged or slightly elevated levels of antioxidants have been noted [7]–[11]. Even though such information is available for whole leaves and for some cell compartments in tomato leaves (e.g. chloroplasts, peroxisomes, mitochondria) the situation in other cell compartments such as nuclei, vacuoles and the cytosol remains unclear [7], [9], [10].

Thus, the aim of this study was to investigate the compartment-specific accumulation of H_2_O_2_ during *Botrytis cinerea* infection in Arabidopsis Col-0 on a high resolution and to correlate it with subcellular ascorbate and glutathione contents.

## Materials and Methods

### Plant material, Botrytis inoculation and sample harvesting

After stratification for 4 d at 4°C seeds of *Arabidopsis thaliana* [L.] Heynh. ecotype Columbia (Col-0) were grown on “Naturahum” potting soil (Ostendorf Gärtnereierden GmbH., Vechta, Germany) in growth chambers with 8/16 h day/night photoperiod. Day and night temperatures were 22°C and 18°C, respectively, the relative humidity was 60% and the plants were kept at 100% relative soil water content. Light intensity varied between 110 and 140 µmol m^−2^ s^−1^.

Cut leaves of 8 weeks-old *Arabidopsis thaliana* Col-0 were placed in petri dishes containing filter paper wetted with sterile water. A 50 µl drop of a *Botrytis cinerea* (strain: Bo510) spore suspension was delivered onto the abaxial side of each leaf (approximately 1×10^5^ spores ml^−1^). Spores were preincubated in 3 g L^−1^ Gamborg's B5 medium (in 10 mM KH_2_PO_4_ buffer, pH 7) supplemented with 25 mM glucose for 2 h to stimulate germination. Control leaves (CL) were treated likewise but without spores in the medium. Petri dishes were sealed and stored at light conditions described above.

Different parameters were investigated for the IS and for the surrounding leaf tissue, which was defined as AIS. Values were then compared to CL, which had been mock-inoculated. As chloroplasts showed the strongest changes in antioxidative capacity the fine structure of chloroplasts was additionally monitored during the infection period. Samples for the different experiments were harvested at the time of the inoculation (0 h), 12, 24, 48, and 96 hpi with the fungal pathogen *Botrytis cinerea*. Due to preliminary investigations these time points were chosen as clear changes could not be determined at earlier time points and as cells at the IS showed massive cell death at 96 hpi which made it impossible to obtain adequate ultrastructure for investigations at later time points.

### Determination of H_2_O_2_ by diaminobenzidine (DAB) staining

Detection of H_2_O_2_ was performed according to Großkinsky et al. [21] by DAB staining. Therefore, leaves were detached from the plants with a razor blade at the above described time points and immersed over night at room temperature (RT) in 4 ml of an aqueous solution of 1 mg/ml DAB (pH 7; Roth, Karlsruhe, Germany). To de-stain leaves, chlorophyll was extracted by incubation in 70% ethanol. All incubation steps were performed in glass vials in the dark. After chlorophyll extraction photographs were taken with a digital camera (Olympus E330, Olympus, Life and Material Science Europa GmbH, Hamburg, Germany).

### Sample preparation for transmission electron microscopy (TEM) and immunogold labeling

**Figure 1:**
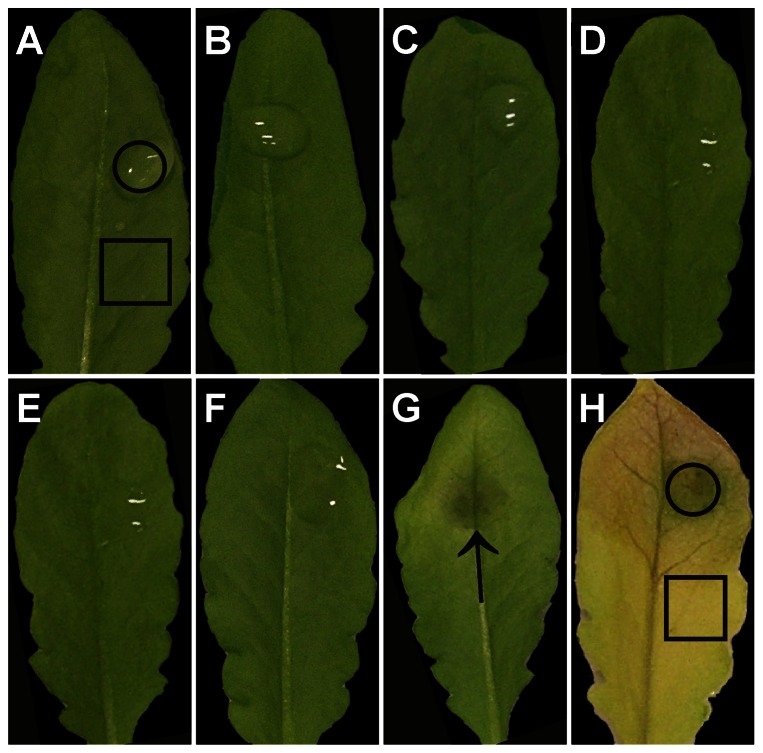
Symptom development on *Arabidopsis thaliana* Col-0 leaves after the inoculation with *Botrytis cinerea*. No symptoms were observed on mock-inoculated controls after 0 h (A), 24 h (B), 48 h (C), and 96 h (D). On infected leaves, visible symptoms remained absent 12 hpi (E) and 24 hpi (F), but developed 48 hpi when one large necrotic area appeared at the IS (arrow in G) and chlorosis developed around this area. Strong symptoms could be observed 96 hpi when the necrotic spot expanded in size and chlorosis appeared on the whole leaf (H). Black frames indicate the areas that were harvested for analysis of ultrastructure and ascorbate or glutathione levels. Square = AIS, circle = IS.

Preparation of samples for TEM and immunogold labeling of ascorbate and glutathione was performed as described previously [22], [23]. Small samples (about 1.5 mm^2^) of at least three different leaves for each treatment and time point (see frames in Figure 1 for approximate harvesting area) were cut on a modeling wax plate either in a drop of i) 2.5% glutardialdehyde in 0.06 M Sørensen phosphate buffer at pH 7.2 for ultrastructural investigations or ii) 2.5% paraformaldehyde, 0.5% glutardialdehyde in 0.06 M Sørensen phosphate buffer at pH 7.2 for cytohistochemical investigations or iii) 5 mM cerium chloride (CeCl_3_) in 50 mM MOPS-buffer (pH 7.2) for subcellular H_2_O_2_ visualization. Samples for ultrastructural and cytohistochemical investigations were then transferred into glass vials and fixed for 90 min at RT in the above mentioned solutions. Samples for the visualization of subcellular H_2_O_2_ distribution were incubated with CeCl_3_ solution for 60 min and then fixed in 2.5% glutardialdehyde in 0.06 M Sørensen phosphate buffer at pH 7.2 for 90 min at RT. For ultrastructural analysis and H_2_O_2_ localization samples were then rinsed in 0.06 M Sørensen phosphate buffer (4 times for 15 min each) and post-fixed in 1% osmium tetroxide in 0.06 M Sørensen phosphate buffer for 90 min at RT. The samples were then dehydrated in a graded series of increasing concentrations of acetone (50%, 70%, 90%, and 100%). Pure acetone was then exchanged for propylene oxide and the specimens were gradually infiltrated with increasing concentrations of Agar 100 epoxy resin (30%, 60%, and 100%) mixed with propylene oxide for a minimum of 3 h per step. Samples were finally embedded in pure, fresh Agar 100 epoxy resin (Agar Scientific Ltd, Stansted, UK) and polymerized at 60°C for 48 h. Ultrathin sections (80 nm) were cut with a Reichert Ultracut S ultramicrotome (Leica Microsystems, Vienna, Austria), post-stained with lead citrate (1% dissolved in 0.6 M NaOH) and uranyl-acetate (2% dissolved in aqua bidest) for 15 min. Sections were then observed in a Philips CM10 TEM. For cytohistochemical investigations samples were rinsed in 0.06 M Sørensen phosphate buffer (pH 7.2) for 4 times 15 min after fixation. They were then dehydrated in increasing concentrations of acetone (50%, 70%, and 90%) at RT for 20 min at each step. Subsequently, specimens were gradually infiltrated with increasing concentrations of LR-White resin (30%, 60% and 100%; London Resin Company Ltd., Berkshire, UK) mixed with acetone (90%) for a minimum of 3 h per step. Samples were finally embedded in pure, fresh LR-White resin and polymerized at 50°C for 48 h in small plastic containers under anaerobic conditions. Ultrathin sections (80 nm) were cut with a Reichert Ultracut S ultramicrotome (Leica Microsystems, Vienna, Austria).

### Determination of compartment-specific H_2_O_2_ accumulation

The accumulation of H_2_O_2_ was determined with a modified method according to Blokhina et al. [24]. Micrographs of randomly photographed sections of at least four different samples stained with CeCl_3_ were digitized and the percentage of areas covered with CeCl_3_ precipitates were measured automatically using the software package Cell D with the particle analysis tool (Olympus, Life and Material Science Europa GmbH, Hamburg, Germany). Measurements were performed in different visually identified and manually traced cell structures such as cell walls, mitochondria, plastids, nuclei and the cytosol. A minimum of 40 sectioned cell structures from at least 15 different cells were evaluated for CeCl_3_-staining. The obtained data were statistically evaluated using Statistica (Stat-Soft Europe, Hamburg, Germany).

### Determination of chloroplast number and their fine structures

Changes in the number of chloroplasts and their inner structures were evaluated according to Zechmann et al. [25] by investigating four different leaf samples for each the IS, AIS and CL. An Olympus AX70 light microscope (Olympus, Life and Material Science Europa GmbH, Hamburg, Germany) with a 40× objective lens (n.a. 0.5–1.35) was used to determine the number of sectioned chloroplasts in the palisade cell layer and the spongy parenchyma by counting the chloroplasts per cell on 4 semithin cross-sections (3 µm) for each replicate sample. A minimum of 100 cells per leaf type were examined to calculate the number of sectioned chloroplasts in the cells. Ultrathin sections were investigated with the TEM to determine changes in the ultrastructure of the chloroplasts including the thylakoid-system, starch grains, and plastoglobuli. These structures were then analyzed as digital images using the program Optimas 6.5.1 (BioScan Corp.). A minimum of 20 sectioned chloroplasts from at least 10 different cells from four different samples per treatment were examined.

### Immunogold labeling of glutathione and ascorbate

Immunogold labeling of glutathione and ascorbate was done according to Zechmann et al. [22], [23] with ultrathin sections on coated nickel grids with the automated immunogold labeling system Leica EM IGL (Leica, Microsystems).The sections were blocked for 20 min with 2% bovine serum albumine (BSA, Sigma-Aldrich, St. Louis, MO, USA) in phosphate buffered saline (PBS, pH 7.2) and then treated with the primary antibody against ascorbate (anti-ascorbate rat polyclonal IgG; Abcam plc, Cambridge, UK) diluted 1∶300 in PBS containing 1% BSA and glutathione (anti-glutathione rabbit polyclonal IgG, Millipore Corp., Billerica, MA, USA) diluted 1∶50 in PBS containing 1% goat serum for 2 h at RT. After a short rinse in PBS (3 times 5 min), samples were incubated with a 10 nm gold-conjugated secondary antibody (goat anti-rabbit IgG for glutathione labeling and goat and rat IgG for ascorbate labeling, British BioCell International, Cardiff, UK) diluted 1∶50 (for sections incubated with the glutathione antibody) and 1∶100 (for sections incubated with the ascorbate antibody) in PBS for 90 min at RT. After a short wash in PBS (3 times 5 min), and distilled water (2 times 5 min) labeled grids were either immediately observed in a Philips CM10 TEM or post-stained with uranyl-acetate (2% dissolved in aqua bidest) for 15 s.

Micrographs of randomly photographed immunogold labeled sections were digitized and gold particles were counted automatically using the software package Cell D with the particle analysis tool (Olympus, Life and Material Science Europa GmbH, Hamburg, Germany) in different visually identified and manually traced cell structures (mitochondria, plastids, nuclei, peroxisomes, the cytosol, vacuoles). For statistical evaluation at least four different samples were examined. A minimum of 20 (peroxisomes and vacuoles) to 60 (other cell structures) sectioned cell structures of at least 15 different cells were analyzed for gold particle density per sample. The obtained data were statistically evaluated using Statistica (Stat-Soft Europe, Hamburg, Germany).

Several negative controls were made to support the specificity of the immunogold procedure. Negative controls were treated either with (i) gold conjugated secondary antibody (goat anti-rat IgG for ascorbate and goat anti-rabbit IgG for glutathione) without prior incubation of the section with the primary antibodies, (ii) non-specific secondary antibody (goat anti-rabbit IgG for ascorbate and goat anti-rat IgG for glutathione), (iii) pre-immune serum instead of the primary antibody and (iv) primary antibody against ascorbate and glutathione pre-adsorbed with an excess of reduced and oxidized ascorbate and glutathione, respectively for 2 h prior to labeling of the sections. For the latter a solution containing either 10 mM of ascorbic acid, dehydroascorbic acid, reduced or oxidized glutathione was incubated with or without 0.5% glutardialdehyde for 1 h. Excess glutardialdehyde was saturated by incubation for 30 min in a solution of 1% (w/v) BSA. The resulting solutions were both used in independent experiments to saturate the anti-ascorbate and glutathione antibodies for 2 h prior to its use in the immunogold labeling procedure described above. Labeling on sections treated as negative controls showed no or only very few gold particles bound to ascorbate and glutathione which was similar to previous results obtained by using the same methods in different plant species [22], [23].

## Results

### Symptom development

Symptom development was evaluated over a time period of 96 hpi. CL did not develop symptoms throughout the time of investigation (Fig. 1A–D). Leaves inoculated with *Botrytis cinerea* did not show symptoms before 48 hpi (Figure 1E–G). Then dark necrotic lesions formed at the IS whereas the AIS showed the beginning of chlorosis (Figure 1G). Necrosis and chlorosis increased in size 96 hpi when the whole leaf turned yellow and one large necrotic spot developed roughly covering the IS (Figure 1H). On the light microscopical level fungal hyphae were observed first at 48 hpi on the surface of the leaves at the IS (Figure S1). 96 hpi hyphae could be observed throughout the degenerated leaf tissue at the IS which consisted of dead cells with disintegrated cell compartments only (Figure S1).

### H_2_O_2_ localization by DAB and CeCl_3_ staining

**Figure 2:**
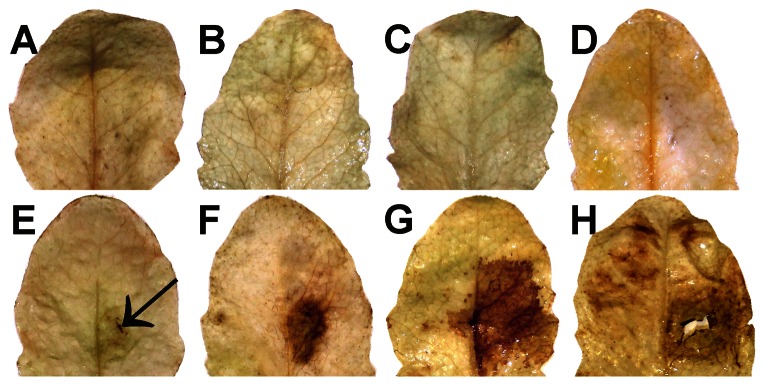
Visualization of H_2_O_2_ by DAB staining. No staining was observed on mock-inoculated controls after 0 h (A), 24 h (B), 48 h (C), and 96 h (D). First staining of H_2_O_2_ in the form of small local spots (arrows) at the IS could be observed 12 hpi (E). Stained areas increased in size and fully covered the IS at 24 hpi (F). H_2_O_2_ staining reached its maximum at 48 hpi when it could be observed at the IS and the AIS (G). Staining of H_2_O_2_ at the IS and AIS could also be observed at 96 hpi (H).

H_2_O_2_ did not accumulate in leaves of mock-inoculated control plants over the period of investigation (Figure 2A–D). DAB staining revealed the accumulation of H_2_O_2_ in the form of small brown stained areas at 12 hpi at the IS (Figure 2E). At this time point H_2_O_2_ staining was not observed at the AIS. H_2_O_2_ accumulation at 24 hpi increased in size and fully covered the IS at 24 hpi (Figure 2F). H_2_O_2_ staining reached its maximum at 48 hpi when it could be observed at the IS and the AIS (Figure 2G). Staining of H_2_O_2_ at the IS and AIS could also be observed at 96 hpi but was weaker when compared to the other time points (Figure 2H).

**Figure 3:**
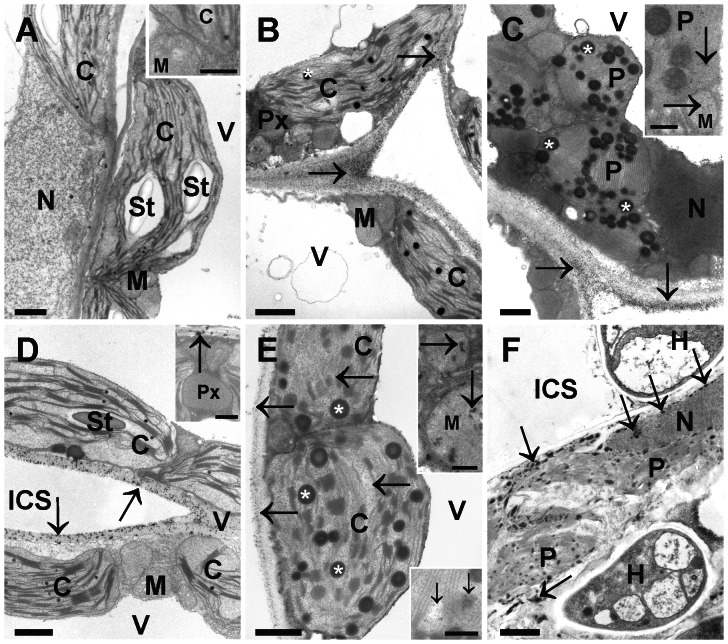
Visualization of H_2_O_2_ by CeCl_3_ staining. No staining was observed in cells of mock-inoculated controls (A: 96 hpi). At the AIS H_2_O_2_ accumulation (arrows) was detected within cell walls 48 hpi (B) and inside mitochondria (M) and plastids (P) 96 hpi (inset in C). Note that plastoglobuli (white asterisks) within chloroplasts (C) and plastids (P) increased in size with advancing infection (best seen in B and E when compared to A). At the IS small dark precipitates of H_2_O_2_ staining (arrows) were observed 24 hpi (D) in cell walls (arrows) but not in the other cell compartments and 48 hpi (E) in cell walls, in the matrix of mitochondria (M, inset upper right corner) and stroma of chloroplasts (inset lower right corner). Dark precipitates (arrows) were observed at the IS throughout the cytoplasm of leaf cells 96 hpi (F). At the IS 96 hpi only remnants of organelles such as plastids (P) and nucleus (N) could be found in the condensed cytoplasm. H = hyphae, ICS = intercellular spaces, Px = peroxisomes, St = starch, V = vacuoles. Bars in inset of A, C, and E = 0.25 µm, D = 0.5 µm. Bars in all other images 1 µm.

CeCl_3_ staining showed that H_2_O_2_ did neither accumulate in controls (Figure 3A) nor in plants inoculated with the fungus until 24 hpi (data not shown). First signs of H_2_O_2_ accumulation in cell walls were observed at the IS 24 hpi (Figure 3D) and at the AIS 48 hpi (Figure 3B). At the IS 48 hpi H_2_O_2_ accumulation was obvious in the cell walls and inside mitochondria and chloroplasts (Figure 3E). Chloroplasts at this stage contained large plastoglobuli when compared to the controls, which displayed only small plastoglobuli visible as small dark spots in the stroma. At the IS 96 hpi leaves were penetrated by fungal hyphae which grew inter- and intracellulary. Plant cells at this stage contained no vacuoles, a condensed cytoplasm with remnants of plastids, nuclei and mitochondria (Figure 3F). Throughout the cytoplasm black CeCl_3_ precipitates indicated accumulation of H_2_O_2_ (Figure 3F). At the AIS H_2_O_2_ accumulation could be observed 96 hpi inside the cell walls and within chloroplasts and mitochondria (Figure 3C). Plastids at this stage contained large plastoglobuli and only single thylakoids in the dense stroma. Thus, the development of intracellular symptoms in the AIS was delayed by 2 d compared to the IS.

**Figure 4:**
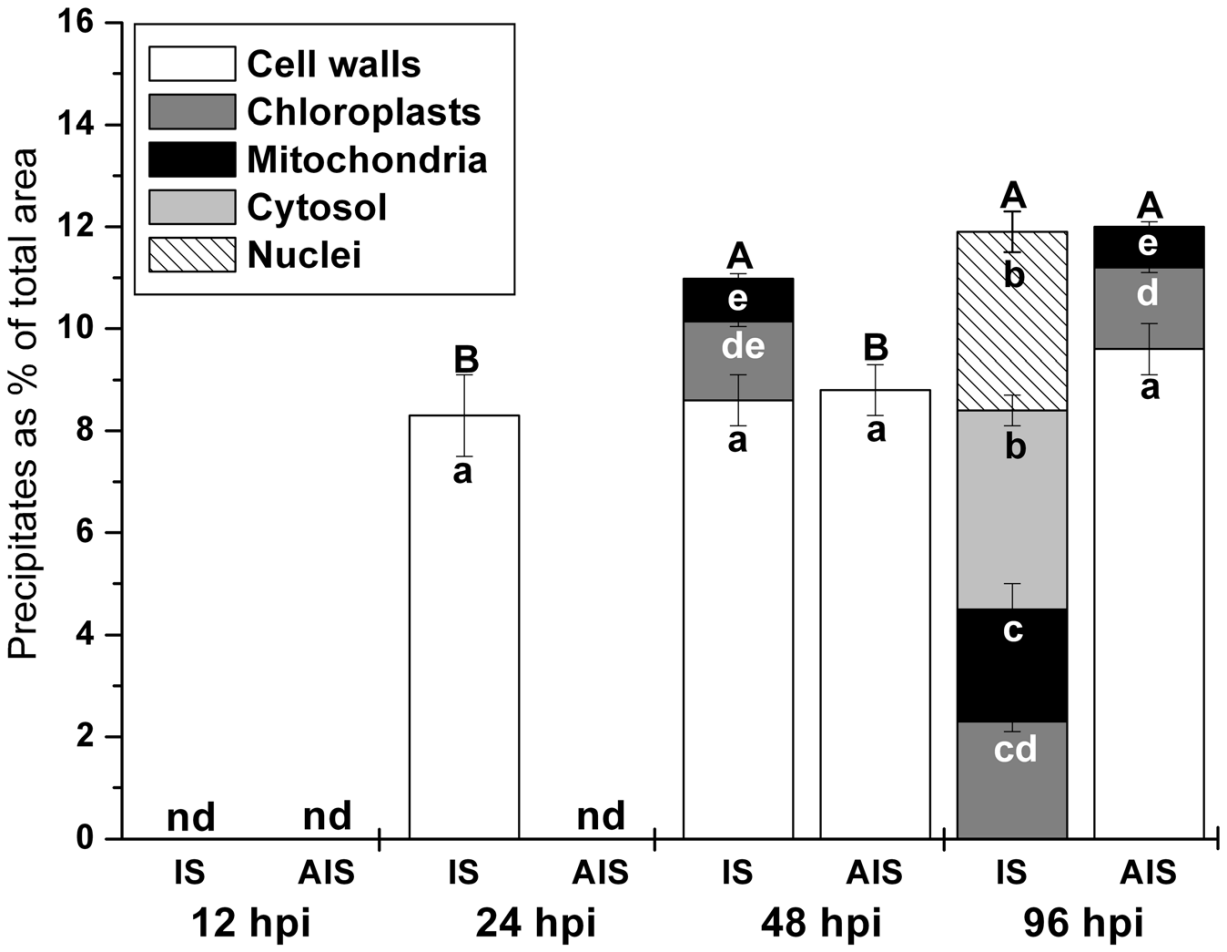
Compartment-specific accumulation of H_2_O_2_. The subcellular accumulation of H_2_O_2_ was quantified on transmission electron micrographs by determining the area of CeCl_3_ staining in the different cell compartments. Measurements were performed 12, 24, 48 and 96 hpi at the IS and the AIS. Graphs show means with standard errors which document the percentage of areas covered by CeCl_3_ precipitates in the individual cell compartments. n>40 for the individual cell compartments. Different lowercase letters indicate significant differences (p<0.05) between the individual cell compartments whereas uppercase letters indicate significant differences between the total amount of CeCl_3_ staining for all analyzed cell compartments taken together. Data were analyzed with the Kruskal-Wallis test followed by post-hoc comparison according to Conover. nd = not detected.

Quantification of the area covered by CeCl_3_ precipitates revealed a steady increase of H_2_O_2_ accumulation (Figure 4). Interestingly, the response at the AIS at 48 hpi and 96 hpi mimicked the response of the IS at 24 hpi and 48 hpi, respectively (Figure 4). When present CeCl_3_ precipitates covered around 10% of the cell walls. CeCl_3_ precipitates covered about 1.5% of the chloroplasts at the IS at 48 hpi and the AIS at 96 hpi, and 2.3% at the IS at 96 hpi (Figure 4). In mitochondria an area of about 0.8% was covered by CeCl_3_ precipitates at the IS at 48 hpi and at the AIS at 96 hpi, and 2.6% at the IS at 96 hpi. CeCl3 precipitates in nuclei and the cytosol where only found at the IS at 96 hpi where they covered 3.5% and 3.9%, respectively (Figure 4).

### Ascorbate labeling

**Figure 5:**
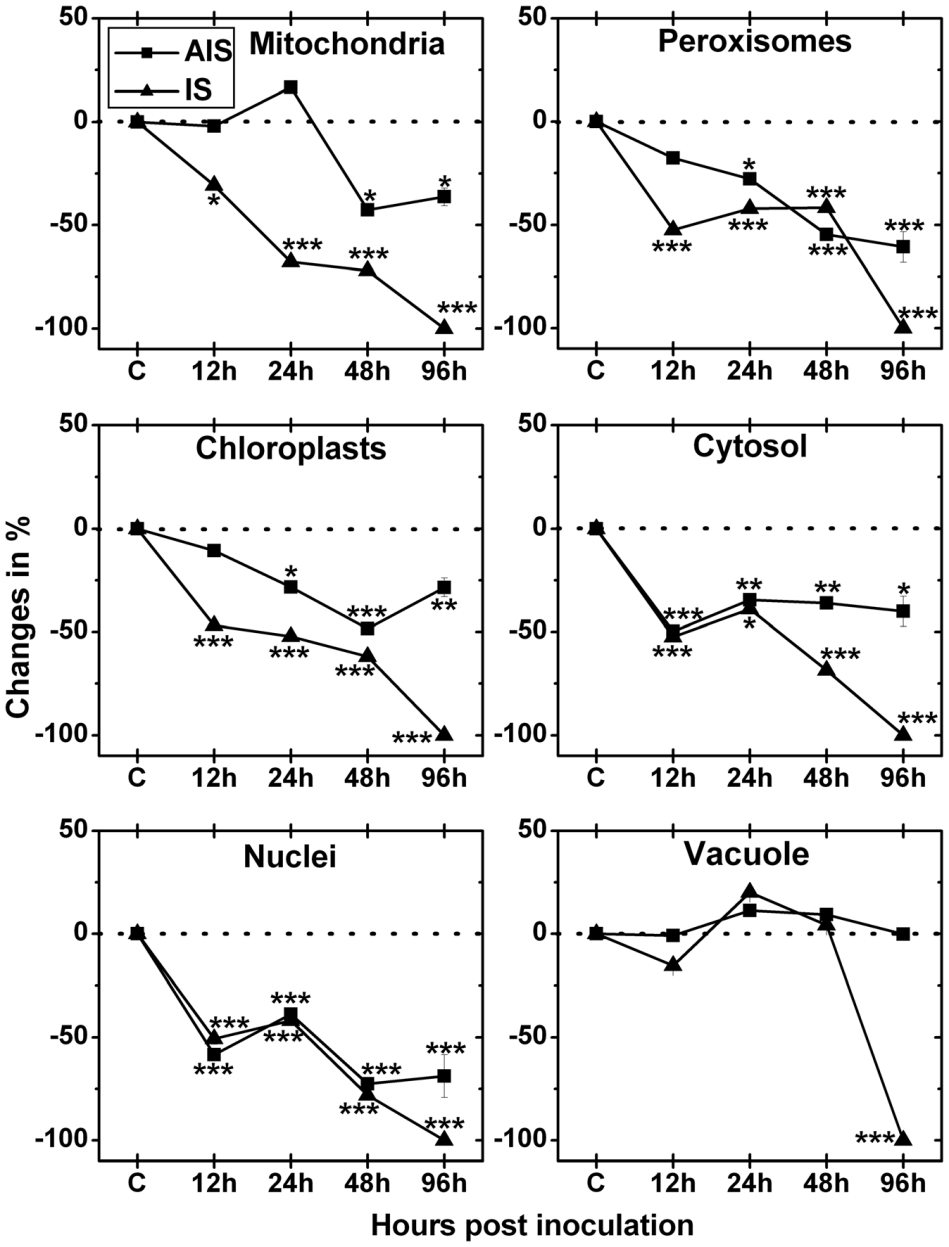
Compartment-specific changes in ascorbate labeling. Labeling was evaluated within leaf mesophyll cells of *Arabidopsis thaliana* Col-0 inoculated with *Botrytis cinerea* and compared to CL. Measurements were performed 0 h ( = CL, represented by the dotted line), 12, 24, 48 and 96 hpi at the IS and the AIS. Shown are means with standard errors and changes in the amount of gold particles bound to ascorbate per µm^2^ in the respective cell compartment. n>20 for peroxisomes and vacuoles and n>60 for other cell structures. Significant differences were calculated using the Mann-Whitney U-test; *, ** and ***, respectively, indicate significance at the 0.05, 0.01 and 0.001 levels of confidence. Square = AIS; triangle = IS.

Subcellular changes of ascorbate labeling were investigated by TEM at 12, 24, 48 and 96 hpi in leaves of *Arabidopsis thaliana* Col-0 plants inoculated with *Botrytis cinerea* (Figure 5, Figure S2, and Figure S3). Ascorbate levels in mock-inoculated control plants did not change significantly over the course of the experiment (data not shown). Therefore, a mean of the subcellular ascorbate content from all time points was calculated (Table S1) for CL and used as standard values to determine differences to *Botrytis cinerea* inoculated plants at 12, 24, 48 and 96 hpi. As ascorbate labeling at the IS at 96 hpi was reduced to background levels (labeling was only achieved in fungal hyphae, see Figure S3D) a 100% decrease was calculated for all cell compartments at this time point (Figure 5). Subcellular ascorbate levels significantly decreased in mitochondria at the IS 12, 24, and 48 hpi (−31%, −68%, −72%, respectively) whereas at the AIS significantly decreased amounts of ascorbate specific labeling could only be observed 48 and 96 hpi (−43% and −36%, respectively) when compared to CL. In chloroplasts, nuclei, peroxisomes and the cytosol decreased amounts of ascorbate specific labeling at the IS were observed 12 hpi (around −50%), 24 hpi (−52% for chloroplasts and approximately −40% for all other compartments), and 48 hpi (−62%, −78%, −42%, −68%, respectively) when compared to CL. At the AIS decreased amounts of gold particles bound to ascorbate were found in chloroplasts and peroxisomes 24 hpi (both −28%), 48 hpi (−48% and −55%, respectively) and 96 hpi (−28% and −61%, respectively) when compared to CL. In nuclei and the cytosol labeling was decreased at the AIS 12 hpi (−58% and −50% , respectively), 24 hpi (−39% and −34% , respectively), 48 hpi (−73% and −36%, respectively), and 96 hpi (−69% and −40%, respectively) when compared to CL (Figure 5).

### Glutathione labeling

**Figure 6:**
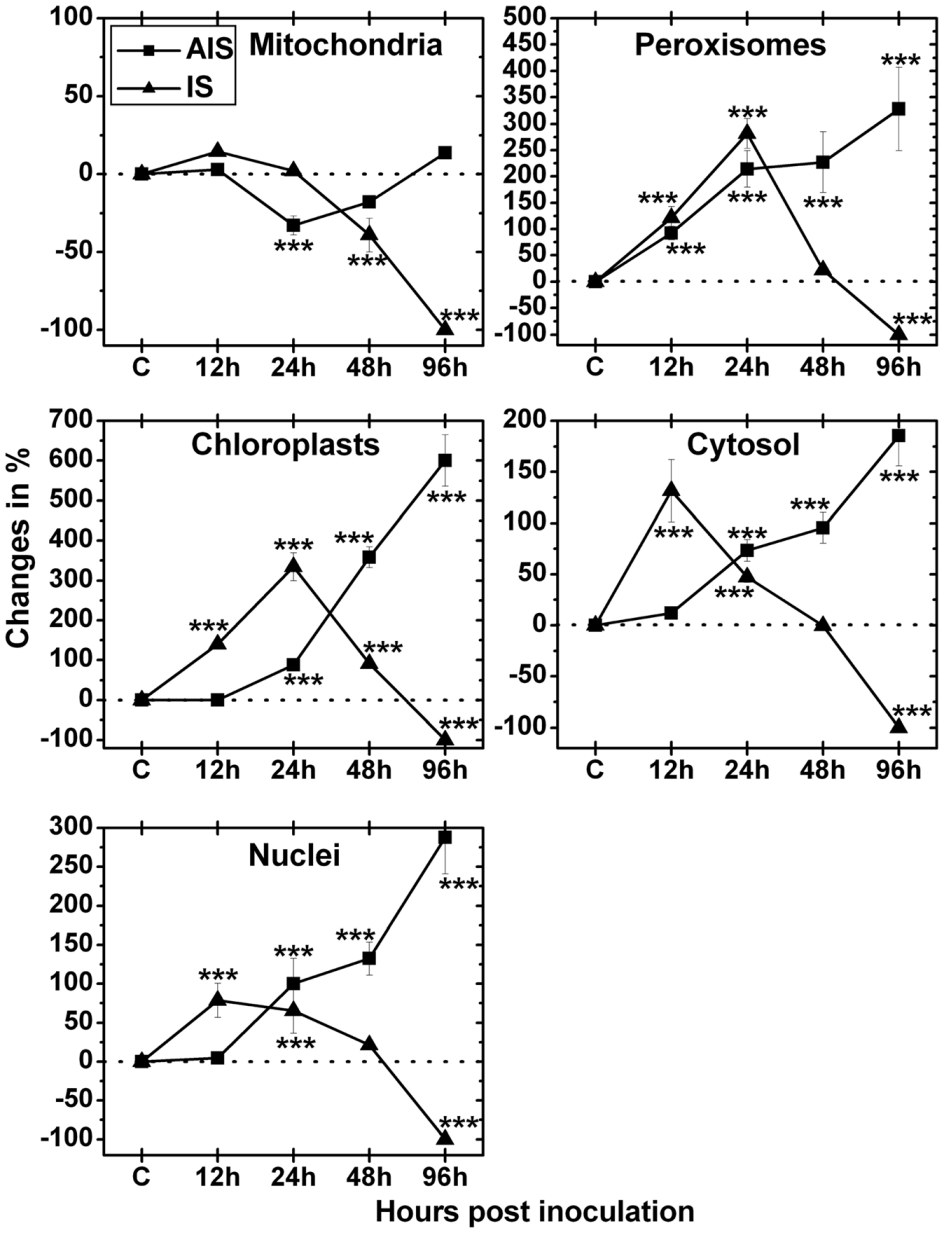
Compartment-specific changes in glutathione labeling. Labeling was evaluated within leaf mesophyll cells of *Arabidopsis thaliana* Col-0 inoculated with *Botrytis cinerea* and compared to CL. Measurements were performed 0 h ( = CL, represented by the dotted line), 12, 24, 48 and 96 hpi at the IS and the AIS. Shown are means with standard errors and changes in the amount of gold particles bound to glutathione per µm^2^ in the respective cell compartment. n>20 for peroxisomes and vacuoles and n>60 for other cell structures. Significant differences were calculated using the Mann-Whitney U-test; *, **, and ***, respectively, indicate significance at the 0.05, 0.01 and 0.001 levels of confidence. Square = AIS; triangle = IS.

Subcellular changes of glutathione labeling were investigated by TEM at 12, 24, 48 and 96 hpi in leaves of *Arabidopsis thaliana* Col-0 plants inoculated with *Botrytis cinerea* (Figure 6, Figure S4, and Figure S5). As with ascorbate, mock-inoculated control plants displayed no different glutathione values at the various time points (data not shown). Therefore, a mean of subcellular glutathione levels was calculated (Table S1) for CL and used as standard to determine differences to *Botrytis cinerea* inoculated plants at 12, 24, 48 and 96 hpi. As glutathione labeling at the IS at 96 hpi was reduced to background levels (labeling was only achieved in fungal hyphae, see Figure S5D) a 100% decrease was calculated for all cell compartments at this time point (Figure 6).

At the IS mitochondria showed a significant decrease in glutathione labeling density at 48 hpi (39%) when compared to CL. In mitochondria glutathione labeling at the AIS was found to be significantly decreased 24 hpi (−33%) but showed no significant changes at other time points when compared to CL. At the IS glutathione labeling in chloroplasts was strongly increased 12 hpi (141%), 24 hpi (335%) and 48 hpi (92%). A similar pattern was found in nuclei, peroxisomes and the cytosol where glutathione labeling density was significantly increased at the IS 12 hpi (79%, 122%, and 132%, respectively), and 24 hpi (65%, 282%, and 47%, respectively), when compared to CL. At the AIS a strong and significant increase of glutathione specific labeling was observed in chloroplasts, nuclei, and the cytosol 24 hpi (88%, 100%, and 73%, respectively), 48 hpi (358%, 132%, and 95%, respectively), and 96 hpi (600%, 288%, and 186%, respectively) when compared to CL. In peroxisomes of the same leaf area density of glutathione labeling was found to be increased 12 hpi (93%), 24 hpi (214%), 48 hpi (227%) and 96 hpi (328%) when compared to CL (Figure 6). Glutathione in vacuoles was found to be very low below or at background levels and was therefore not further evaluated (Figure S4 and Figure S5).

### Chloroplast number and fine structure

**Figure 7:**
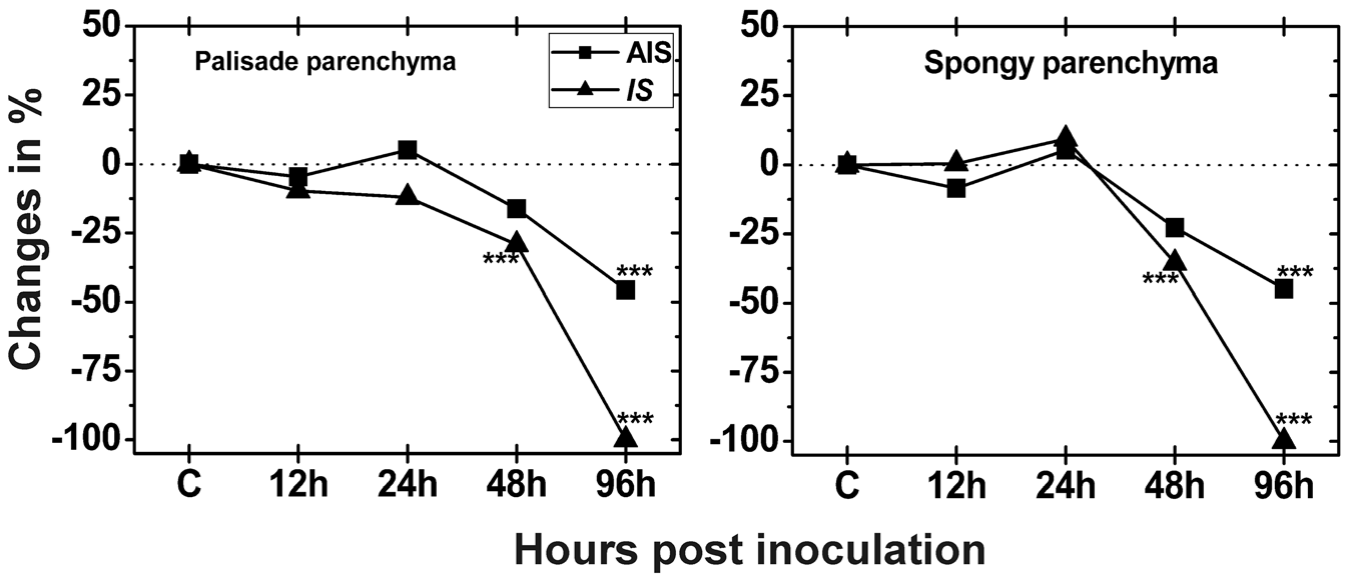
Changes in the relative number of chloroplasts. Number of chloroplasts was evaluated per cell on longitudinal semithin-sections of the palisade cell layer and the spongy parenchyma in *Arabidopsis thaliana* Col-0 leaves inoculated with *Botrytis cinerea* and compared to CL. Measurements were performed 0 h ( = CL, represented by the dotted line), 12, 24, 48 and 96 hpi at the IS and the AIS. Shown are means with standard errors which document changes in the number of chloroplasts. n>100 chloroplasts per treatment. Significant differences were calculated using the Mann-Whitney U-test; *, **, and ***, respectively, indicate significance at the 0.05, 0.01 and 0.001 levels of confidence. Square = AIS; triangle = IS.

**Figure 8:**
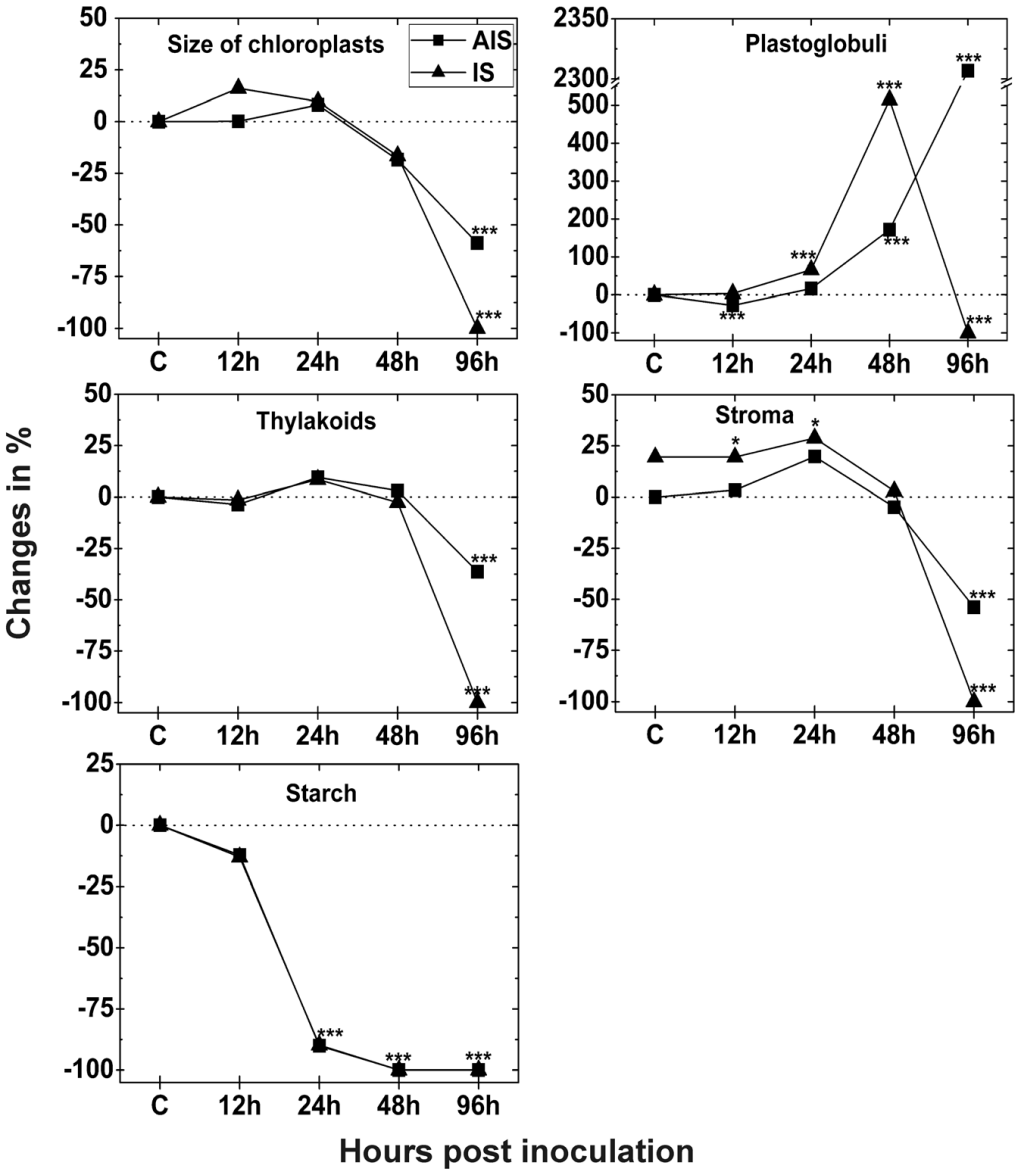
Changes in the size and fine structure of chloroplasts. Size and fine structures of chloroplasts was evaluated by TEM on longitudinal ultrathin sections within the mesophyll of leaves from *Arabidopsis thaliana* Col-0 leaves inoculated with *Botrytis cinerea* and compared to CL. Measurements were performed 0 h ( = CL, represented by the dotted line), 12, 24, 48 and 96 hpi at the IS and the AIS. Shown are means with standard errors. n>100 chloroplasts per treatment. Significant differences were calculated using the Mann-Whitney U-test; *, **, and ***, respectively, indicate significance at the 0.05, 0.01 and 0.001 levels of confidence. Square = AIS; triangle = IS.

Since the number of chloroplasts in control plants did not alter significantly at the different sampling times, a mean of chloroplast number per cell section of all time points was calculated for CL and used as standard level for comparison with *Botrytis cinerea* inoculated plants at 12, 24, 48 and 96 hpi. Mock-inoculated plants contained about 17 and 8 chloroplasts in cell sections of the palisade and spongy parenchyma, respectively. At the IS the number of chloroplasts decreased significantly in cell sections of both tissues 48 hpi (29% and 36%, respectively) when compared to CL (Figure 7). At the AIS the number of chloroplasts decreased significantly only at 96 hpi where 46% and 48% less chloroplasts were detected in the palisade and spongy parenchyma, respectively, when compared to CL (Figure 7). Chloroplast fine structure was investigated by TEM throughout the infection period with *Botrytis cinerea*. Again, control plants did not show differences during the whole experiment (Table S2), while dramatic changes were observed for infected tissue. As only remnants of plastids were present at the IS at 96 hpi the number of chloroplast and fine structure could not be clearly evaluated at this time point. Therefore a 100% decrease was calculated for all cell compartments at this sampling time (Figure 8). At the AIS the size of chloroplasts (−58%) and the amount of thylakoids (−36%) was significantly decreased 96 hpi when compared to CL. Starch content was significantly lowered to a similar extent in chloroplasts at the IS and AIS 24 hpi (about −90%), and 48 hpi. Plastoglobuli area significantly increased at the IS at 24 hpi (66%) and 48 hpi (514%) when compared to CL. At the AIS the area of plastoglobuli significantly decreased at 12 hpi (−27%) and significantly increased at 48 hpi (172%) and 96 hpi (2306%) when compared to CL. Stroma content significantly enlarged at the IS at 12 hpi and 24 hpi (20% and 29%) when compared to CL. A significant decrease of the proportion of the stroma was observed in chloroplasts 96 hpi at the AIS when compared to CL (−54%; Figure 8).

## Discussion

The accumulation of ROS such as H_2_O_2_ around the penetrated cell wall as well as in the plasma membrane has been observed in previous studies during *Botrytis cinerea* infection [10], [17], [18], [20], [26]. The results of this study extend these observations to later time points of infection and reveals cell walls, mitochondria, chloroplasts, nuclei and the cytosol as the main location for H_2_O_2_ accumulation at the IS. Accumulation of H_2_O_2_ at the IS could be observed distinctly first at 24 hpi in cell walls, which then extended to mitochondria and chloroplasts 48 hpi and to the cytosol and nuclei at 96 hpi when only dead cells were found at the IS. At the AIS H_2_O_2_ accumulation was observed within the cell walls first at 48 hpi, even though no visible symptoms occurred at this site and time point, and can thus be considered as an early response of the plant against the invading fungus. When chlorosis spread over the whole leaf at 96 hpi H_2_O_2_, accumulation at the AIS was also observed in chloroplasts and mitochondria, which are both known as the primary site of H_2_O_2_ production in plants [10], [11], [20], [27], [28]. Thus, this study demonstrates that plants react with a local production and accumulation of H_2_O_2_ in cell walls, similar to what has been shown in other studies [18], [20], followed by a severe oxidative burst in mitochondria and chloroplasts during the course of infection with a necrotrophic fungus. At the final stage of infection 96 hpi, when only dead cells could be observed at the IS, H_2_O_2_ accumulation was also found in the cytosol and nuclei which correlates with disintegration of the cytoplasm and necrosis at the IS. The results on the subcellular level are supported by observations in tomato wild type leaves where a strong H_2_O_2_ accumulation could be observed after DAB staining at the IS 48 hpi and spread over larger areas associated with lesion progression [26]. As resistant tomato plants reacted with an accumulation of H_2_O_2_ as early as 8 hpi the authors concluded that a timely production of H_2_O_2_ in epidermis cells at the IS is an important mechanism to prevent spreading of fungal hyphae. Other studies with susceptible tomato infected with *Botrytis cinerea* showed H_2_O_2_ accumulation at the interface of appressorium-like structures and the outer epidermis cell walls as early as 12 hpi and 16 hpi, respectively [18], [20], which was similar to the data observed in this study where only a local accumulation of H_2_O_2_ could be observed at the IS 12 hpi. ROS at this stage seemed to be of fungal origin and produced in mitochondria within the hyphae close to the epidermis cell [20]. In beans inoculated with *Botrytis cinerea* ROS production was additionally visualized within epidermis cells, palisade cells and intercellular spaces as early as 3 hpi. 18 hpi and 24 hpi ROS were mainly observed in cell walls but also inside epidermis cells [20].

ROS like H_2_O_2_ are detoxified by antioxidants and related enzymes. Ascorbate and glutathione are the most important antioxidants in plants [12]–[14]. They occur in all cell compartments where they detoxify ROS or H_2_O_2_ through the ascorbate-glutathione cycle [22], [23], [29]. In this study the accumulation of H_2_O_2_ correlated well with a decrease in ascorbate contents. In general, we observed a strong drop in ascorbate levels in all cell compartments (except vacuoles) throughout the infection. At the IS this drop occurred earlier and was more severe than at the AIS showing a good correlation with the accumulation of H_2_O_2_. Similar results have been obtained by electron paramagnetic resonance within Arabidopsis leaves where a massive depletion of ascorbic acid correlated with an increase in ROS [11]. Similar to this study the authors could also demonstrate that ROS accumulation was higher at the IS (rotted tissue), followed by chlorotic areas and green areas of the infected leaf. In other studies performed on tomato infected with *Botrytis cinerea* the decrease of ascorbate contents, insufficient activities of related antioxidative enzymes and a general shift towards the oxidative state at the cellular and organelle level was related to disease progress [7]–[10]. The findings of these earlier studies and the reduced ascorbate contents and the accumulation of H_2_O_2_ observed here prove that the decreasing capacity of the antioxidative system triggers an oxidative burst leading to chlorosis and eventually cell death during susceptible *Botrytis cinerea* infection in Arabidopsis. This conclusion is supported by changes in glutathione contents at the IS which showed a strong increase 12 and 24 hpi followed by a strong drop 48 and 96 hpi when H_2_O_2_ accumulation reached its maximum. Whereas subcellular glutathione contents 96 hpi are similar to what has been reported previously for tomato [7]–[10] our findings differ from these studies for early stages of infection. There, peroxisomes and mitochondria showed decreased levels of glutathione starting at 24 hpi and 48 hpi, respectively, and chloroplasts showed similar levels to the control up to 48 hpi [7]–[10]. The reasons for the differences between these previous studies and our results remain unclear but might be explained by the different methods used to measure glutathione contents (cytohistochemistry vs. organelle isolation and biochemical measurement), differences in sampling procedures (different leaf areas vs. whole leaf) and by differences in symptom development, infection and defense processes between Arabidopsis and tomato. In Arabidopsis, for example, a strong up-regulation of genes encoding enzymes responsible for glutathione synthesis has been described recently by high resolution temporal transcriptomic analysis 48 hpi with *Botrytis cinerea* [30]. These results fit well with the general increase of glutathione contents observed in this study in most cell compartments at the IS during the first stages of the infection. In conclusion, from the data shown in this and previous studies it seems that the increase of glutathione metabolism at the beginning of the infection process reflects the increased need of the plant for protection against oxidative stress. Furthermore, it is apparent that the breakdown of the antioxidative system at later stages of infection correlated with the accumulation of ROS which results in the development of chlorosis, necrosis and eventually cell death.

Generally, the response of the AIS lagged behind that of the IS. While H_2_O_2_ accumulation could be observed by DAB and CeCl_3_ staining at the IS at 24 hpi similar effects could be found at the AIS at 48 hpi. Additionally, the situation of H_2_O_2_ accumulation at the AIS at 96 hpi mimicked the situation at the IS at 48 hpi. Similar effects of the antioxidative response could be observed at these time points as ascorbate contents dropped earlier at the IS when compared to the AIS in most cell compartments. In addition, both the initial increase and the drop of glutathione contents at the final stages of infection happened earlier at the IS than at the AIS in most cell compartments. The reason for the delayed response of the AIS can be explained by delayed symptom development as the fungal hyphae have to establish the infection at the IS first before they can spread into other areas of the AIS. Thus, we can conclude that H_2_O_2_ accumulation and the antioxidative response of the AIS lagged behind but mimicked the situation at the IS.

On the subcellular level it is remarkable that the AIS showed a massive accumulation of glutathione in all cell compartments of up to 600% in chloroplasts at 96 hpi when glutathione contents dropped to background levels at the IS. Again, this increase in glutathione contents can be correlated with the up-regulation of genes encoding enzymes responsible for glutathione synthesis found in Arabidopsis infected with *Botrytis cinerea* [30]. Nevertheless, this massive increase in glutathione did not prevent the accumulation of H_2_O_2_ in chloroplasts at the AIS at this time point. This could be due to a shift from reduced to oxidized glutathione despite higher activity of glutathione reductase which was demonstrated for chloroplasts in *Botrytis* infected tomato plants at this time point and could also be observed in whole leaves [9]. The up-regulation of genes encoding enzymes responsible for the reduction of oxidized glutathione has also been observed in Arabidopsis during *Botrytis cinerea* infection [30]. Thus, these results indicate that despite the strong increase of glutathione contents at the AIS the antioxidative system of the plant failed to prevent the accumulation of H_2_O_2_ leading to oxidative damage visible in the form of chlorosis at the AIS and subsequently necrosis caused by massive cell dead at the IS 96 hpi.

Infection of Arabidopsis with *Botrytis cinerea* also induced severe changes in chloroplast fine structure. Interestingly, plastoglobuli contents were massively increased 24 hpi and 48 hpi at the IS and 48 hpi and 96 hpi at the AIS. These results correlate well with a strong decrease in thylakoid content 96 hpi at the AIS. Plastoglobuli are considered to be an important storage subcompartment of degenerated thylakoid membranes [31], to play an important role in the breakdown of carotenoids [32], and to be a reservoir for excess amounts of plastoquinone-9, a-tocopherol and possibly other lipids which cannot be stored in thylakoids [33]. A raise in plastoglobuli number was commonly observed in degrading chloroplasts during senescence [34]–[37] and environmental stress conditions [24], [33], [37]–[42]. Thus, it seems likely that the observed accumulation of H_2_O_2_ inside the chloroplasts triggered thylakoid degradation which resulted in a strong increase in plastoglobuli size until complete disintegration of chloroplasts.

## Conclusions

Summing up, this study demonstrates that ROS production during *Botrytis cinerea* interaction with susceptible Arabidopsis plants induced a severe oxidative bust starting in cell walls at 24 hpi at the IS and spread to mitochondria, chloroplasts, nuclei and the cytosol at later stages. Cell walls, mitochondria and chloroplasts seem to be the main origin of H_2_O_2_ as an accumulation occurred in these cell compartments much earlier than in the cytosol and nuclei. Early responses (time points before 24 hpi) observed in other plant species in previous studies seem to be rather local either triggered by oxidative burst in epidermis cells or by ROS produced by the fungal hyphae. The breakdown of the antioxidative system indicated by a strong drop in ascorbate and glutathione levels at later stages of infection correlated with the accumulation of ROS which resulted in the development of chlorosis, necrosis and eventually cell death and was associated with severe changes in chloroplast structure and number.

## Supporting Information

Figure S1 **Light microscopical images of leaf sections from** ***Arabidopsis thaliana*** **Col-0 inoculated with** ***Botrytis cinerea*** **.** Images in A and D show sections of CL at the beginning (0 h) and at the end of the experiment (96 h), respectively, at the mock-inoculation site. Images in B, C and E, F show leaves at the AIS and at the IS, respectively, at 48 hpi (B, E) and 96 hpi (C, F). Whereas fungal structures remain absent in sections of the AIS (B = 48 hpi, C = 96 hpi) hyphae (arrows) on top (48 hpi) and inside (96 hpi) the leaves could be found at the IS. Bars = 50 µm.
(TIF)

Figure S2 **Transmission electron micrographs showing ascorbate specific labeling at the AIS.** Gold particles bound to ascorbate were detected at the AIS in leaf sections from *Arabidopsis thaliana* Col-0 0 h (A), 24 hpi (B), 48 hpi (C) and 96 hpi (D) with *Botrytis cinerea*. Bars = 1 µm. C = chloroplasts with or without starch (St), CW = cell walls, ICS = intercellular spaces, M = mitochondria, N = nuclei, Px = peroxisomes, V = vacuoles.
(TIF)

Figure S3 **Transmission electron micrographs showing ascorbate specific labeling at the IS.** Gold particles bound to ascorbate were detected at the IS in leaf sections from *Arabidopsis thaliana* Col-0 0 h (A), 24 hpi (B), 48 hpi (C) and 96 hpi (D) with *Botrytis cinerea*. Note that 96 hpi fungal hyphae (H) containing gold particles bound to ascorbate and only remnants of organelles such as plastids (P) could be found in the degenerated leaf cells (D). Bars = 1 µm. C = chloroplasts with or without starch (St), M = mitochondria, N = nuclei, Px = peroxisomes, V = vacuoles.
(TIF)

Figure S4 **Transmission electron micrographs showing glutathione specific labeling at the AIS.** Gold particles bound to glutathione were detected at the AIS in leaf sections from *Arabidopsis thaliana* Col-0 0 h (A), 24 hpi (B), 48 hpi (C) and 96 hpi (D) with *Botrytis cinerea*. Bars = 1 µm. C = chloroplasts with or without starch (St), CW = cell walls, ICS = intercellular spaces, M = mitochondria, N = nuclei, Px = peroxisomes, V = vacuoles.
(TIF)

Figure S5 **Transmission electron micrographs showing glutathione specific labeling at the IS.** Gold particles bound to glutathione were detected at the IS in leaf sections from *Arabidopsis thaliana* Col-0 0 h (A), 24 hpi (B), 48 hpi (C) and 96 hpi (D) with *Botrytis cinerea*. Note that 96 hpi fungal hyphae (H) containing gold particles bound to glutathione and only remnants of organelles such as plastids (P) could be found in the degenerated leaf cells (D). Bars = 1 µm. C = chloroplasts with or without starch (St), CW = cell walls, M = mitochondria, N = nuclei, Px = peroxisomes, V = vacuoles.
(TIF)

Table S1 **Total number of gold particles bound to ascorbate and glutathione in mock inoculated *Arabidopsis thaliana*.** Values are means with standard errors and document the total amount of gold particles bound to ascorbate and glutathione per µm^2^ in different cell compartments of mock inoculated *Arabidopsis thaliana* [L.] Heynh. ecotype Columbia (Col-0). n.d. = not detected. n>20 for peroxisomes and vacuoles and n>60 for other cell structures. Different lowercase letters indicate significant differences (P<0.05) analyzed with the Kruskal-Wallis test followed by post-hoc comparison according to Conover.
(TIF)

Table S2 **Total number and size of chloroplast fine structures in mock inoculated *Arabidopsis thaliana* Col 0.** Values are means with standard errors and document the relative area of internal chloroplast structures and the size of chloroplast in µm^2^ detected by TEM on a longitudinal ultrathin section within the mesophyll of mock inoculated leaves in *Arabidopsis thaliana* [L.] Heynh. ecotype Columbia (Col-0). Different lowercase letters indicate significant differences (P<0.05) analyzed with the Kruskal-Wallis test followed by post-hoc comparison according to Conover. N>20 from at least four different samples.
(TIF)
