# Supplementary figures and images for: High Resolution Imaging of Temporal and Spatial Changes of Subcellular Ascorbate, Glutathione and H2O2 Distribution during Botrytis cinerea Infection in Arabidopsis
Source: PLoS One. 2013 Jun 5;8(6):e65811. doi: 10.1371/journal.pone.0065811 (PMC3673919; doi:10.1371/journal.pone.0065811)

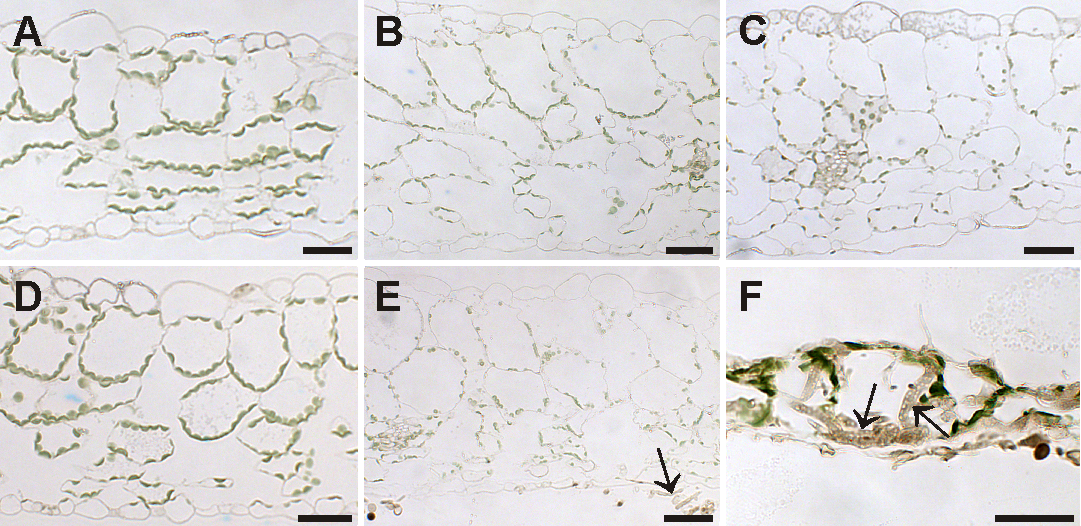

Supplement: Figure S1 — Light microscopical images of leaf sections from Arabidopsis thaliana Col-0 inoculated with Botrytis cinerea . Images in A and D show sections of CL at the beginning (0 h) and at the end of the experiment (96 h), respectively, at the mock-inoculation site. Images in B, C and E, F show leaves at the AIS and at the IS, respectively, at 48 hpi (B, E) and 96 hpi (C, F). Whereas fungal structures remain absent in sections of the AIS (B = 48 hpi, C = 96 hpi) hyphae (arrows) on top (48 hpi) and inside (96 hpi) the leaves could be found at the IS. Bars = 50 µm. (TIF) [file pone.0065811.s001.tif]

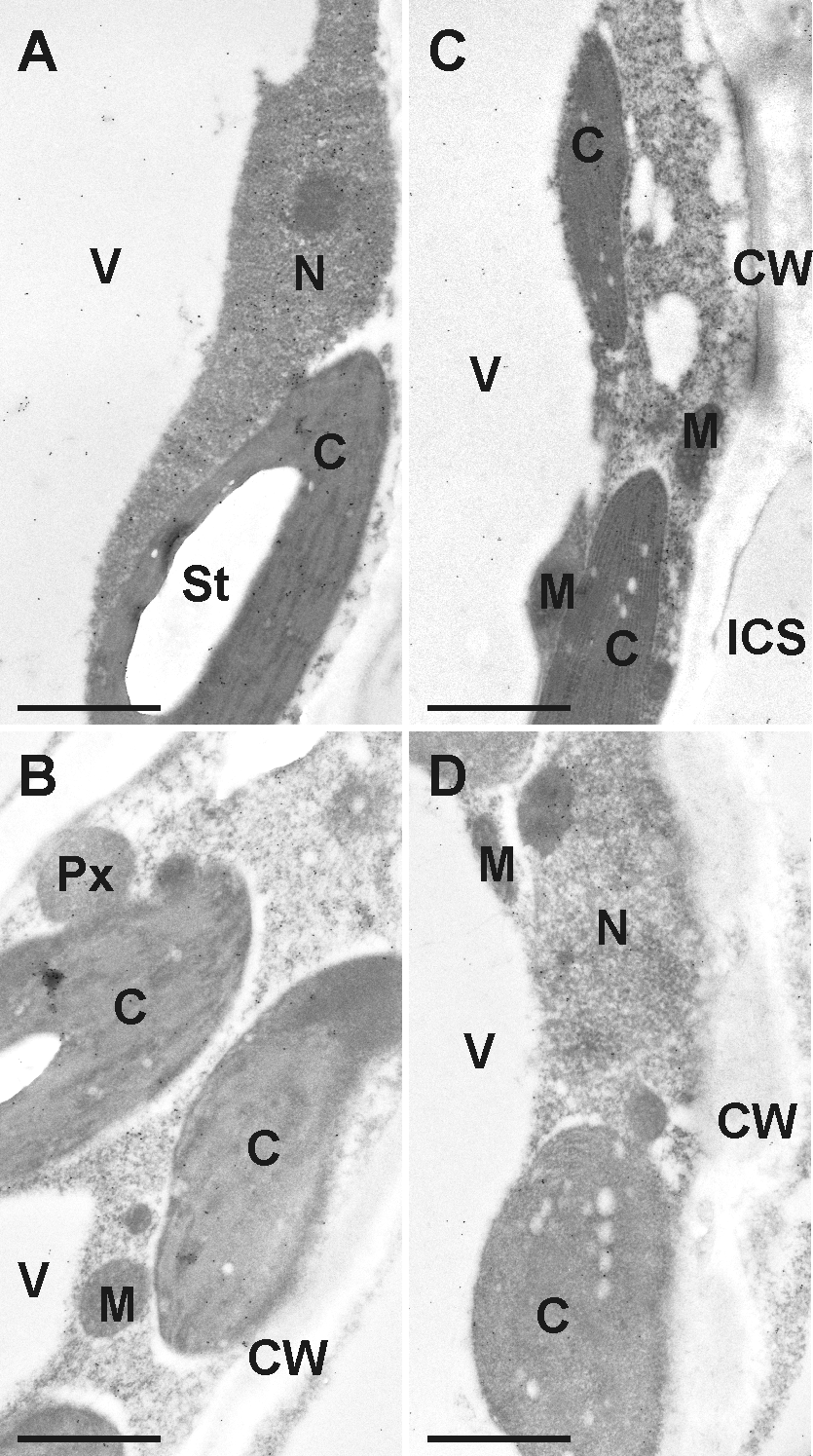

Supplement: Figure S2 — Transmission electron micrographs showing ascorbate specific labeling at the AIS. Gold particles bound to ascorbate were detected at the AIS in leaf sections from Arabidopsis thaliana Col-0 0 h (A), 24 hpi (B), 48 hpi (C) and 96 hpi (D) with Botrytis cinerea. Bars = 1 µm. C = chloroplasts with or without starch (St), CW = cell walls, ICS = intercellular spaces, M = mitochondria, N = nuclei, Px = peroxisomes, V = vacuoles. (TIF) [file pone.0065811.s002.tif]

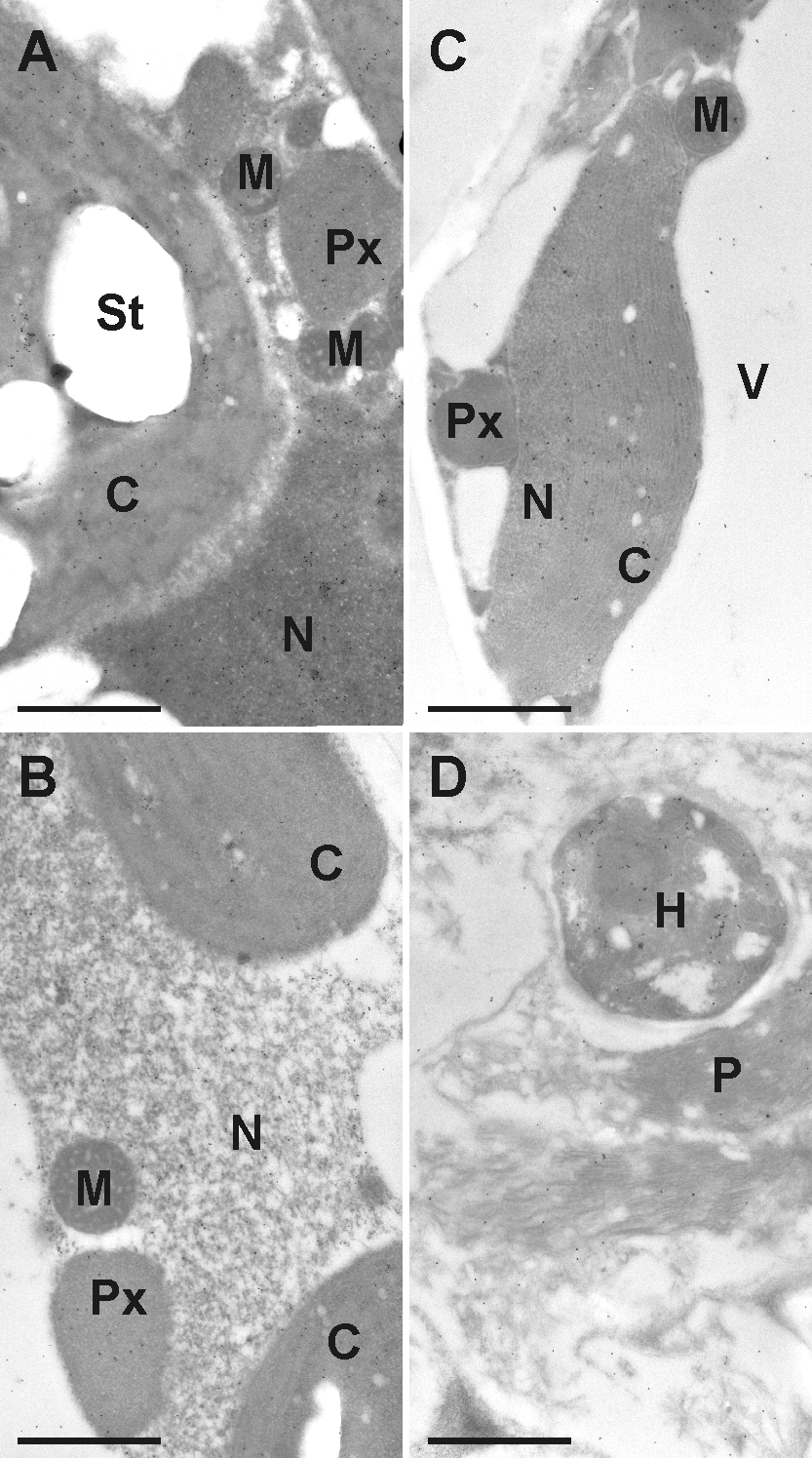

Supplement: Figure S3 — Transmission electron micrographs showing ascorbate specific labeling at the IS. Gold particles bound to ascorbate were detected at the IS in leaf sections from Arabidopsis thaliana Col-0 0 h (A), 24 hpi (B), 48 hpi (C) and 96 hpi (D) with Botrytis cinerea. Note that 96 hpi fungal hyphae (H) containing gold particles bound to ascorbate and only remnants of organelles such as plastids (P) could be found in the degenerated leaf cells (D). Bars = 1 µm. C = chloroplasts with or without starch (St), M = mitochondria, N = nuclei, Px = peroxisomes, V = vacuoles. (TIF) [file pone.0065811.s003.tif]

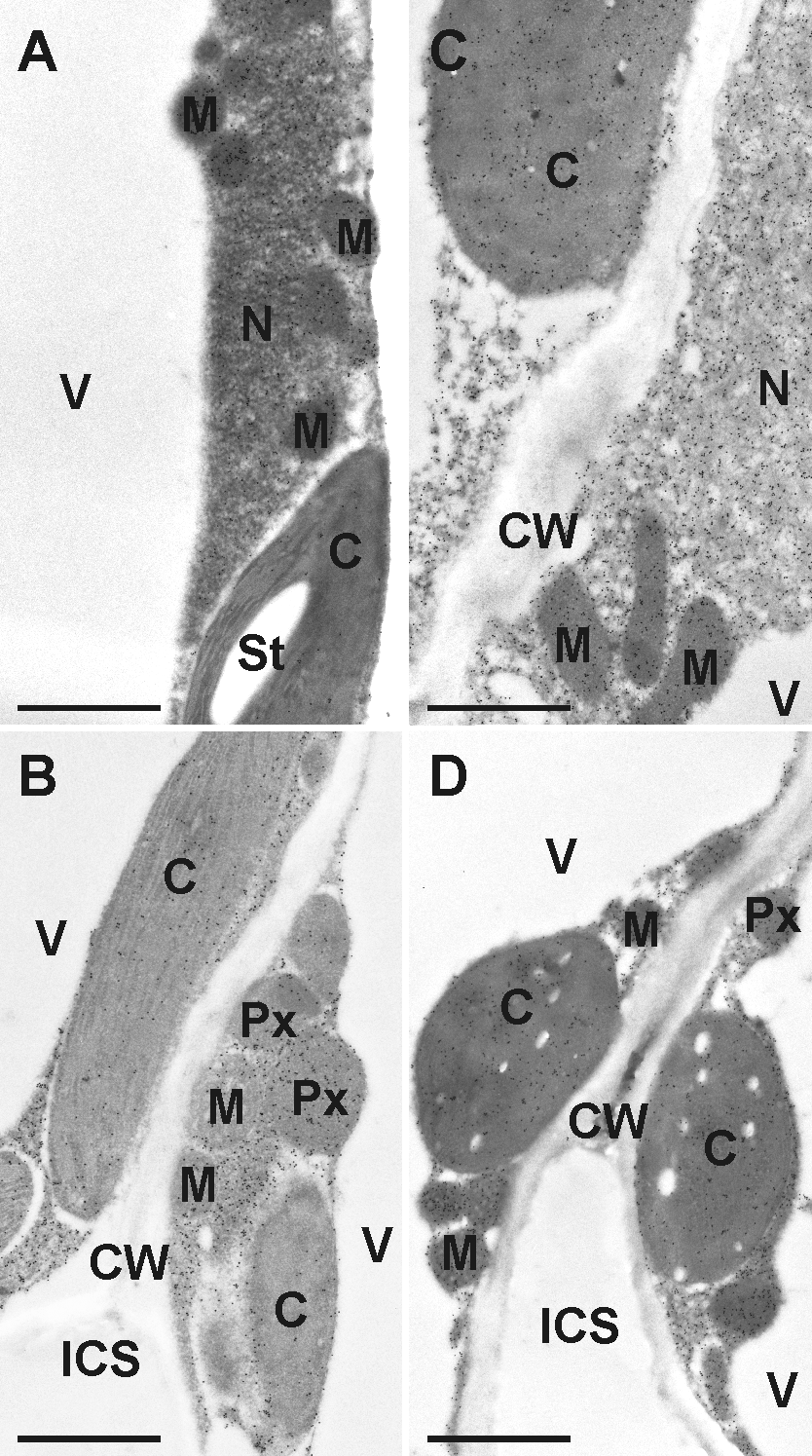

Supplement: Figure S4 — Transmission electron micrographs showing glutathione specific labeling at the AIS. Gold particles bound to glutathione were detected at the AIS in leaf sections from Arabidopsis thaliana Col-0 0 h (A), 24 hpi (B), 48 hpi (C) and 96 hpi (D) with Botrytis cinerea. Bars = 1 µm. C = chloroplasts with or without starch (St), CW = cell walls, ICS = intercellular spaces, M = mitochondria, N = nuclei, Px = peroxisomes, V = vacuoles. (TIF) [file pone.0065811.s004.tif]

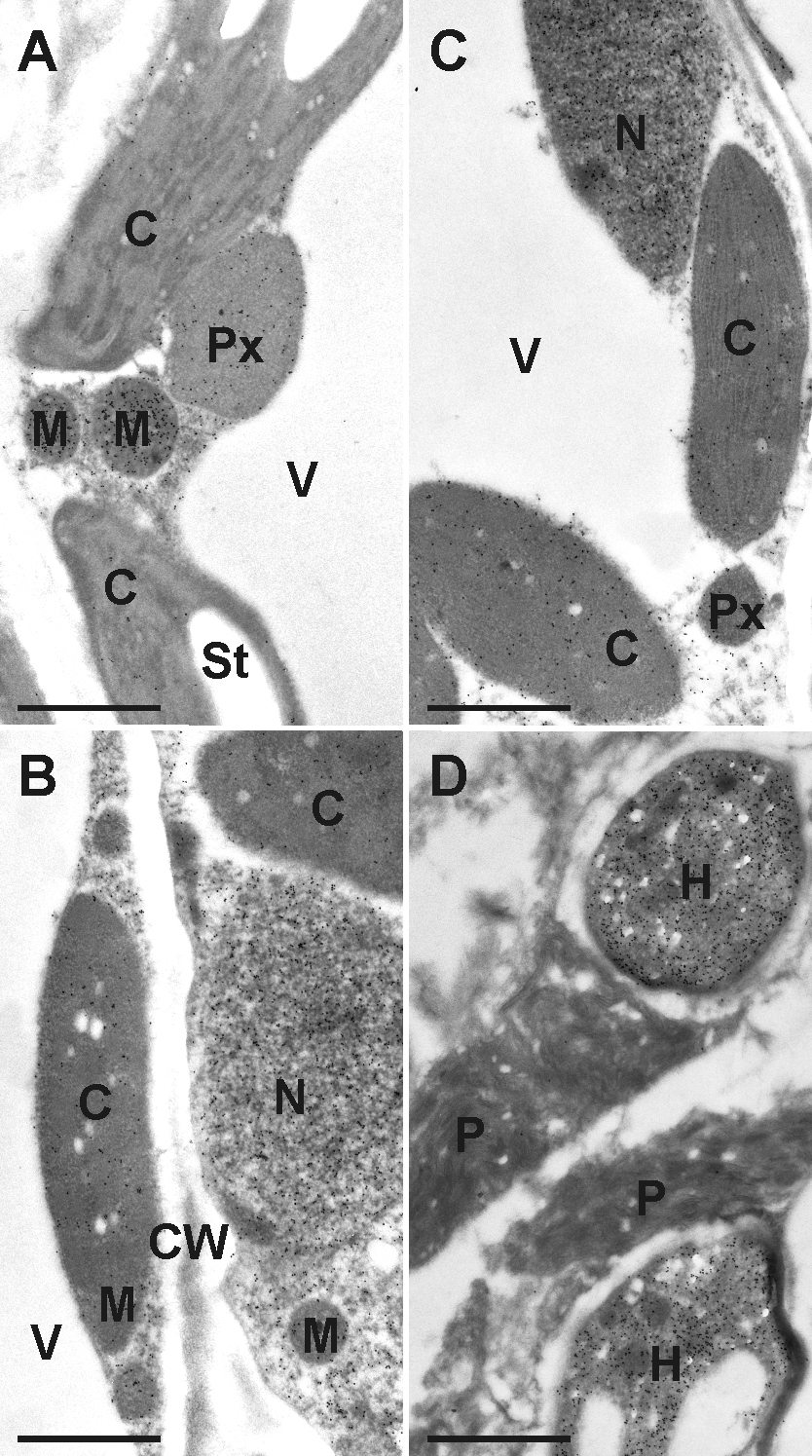

Supplement: Figure S5 — Transmission electron micrographs showing glutathione specific labeling at the IS. Gold particles bound to glutathione were detected at the IS in leaf sections from Arabidopsis thaliana Col-0 0 h (A), 24 hpi (B), 48 hpi (C) and 96 hpi (D) with Botrytis cinerea. Note that 96 hpi fungal hyphae (H) containing gold particles bound to glutathione and only remnants of organelles such as plastids (P) could be found in the degenerated leaf cells (D). Bars = 1 µm. C = chloroplasts with or without starch (St), CW = cell walls, M = mitochondria, N = nuclei, Px = peroxisomes, V = vacuoles. (TIF) [file pone.0065811.s005.tif]

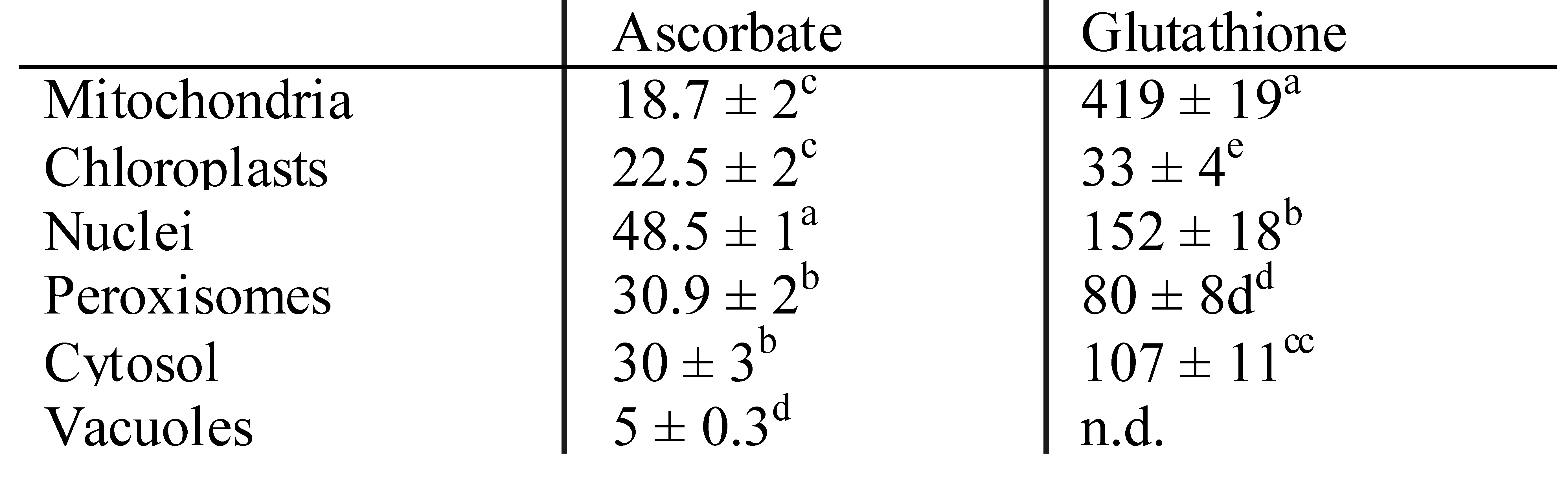

Supplement: Table S1 — Total number of gold particles bound to ascorbate and glutathione in mock inoculated Arabidopsis thaliana. Values are means with standard errors and document the total amount of gold particles bound to ascorbate and glutathione per µm2 in different cell compartments of mock inoculated Arabidopsis thaliana [L.] Heynh. ecotype Columbia (Col-0). n.d. = not detected. n>20 for peroxisomes and vacuoles and n>60 for other cell structures. Different lowercase letters indicate significant differences (P<0.05) analyzed with the Kruskal-Wallis test followed by post-hoc comparison according to Conover. (TIF) [file pone.0065811.s006.tif]

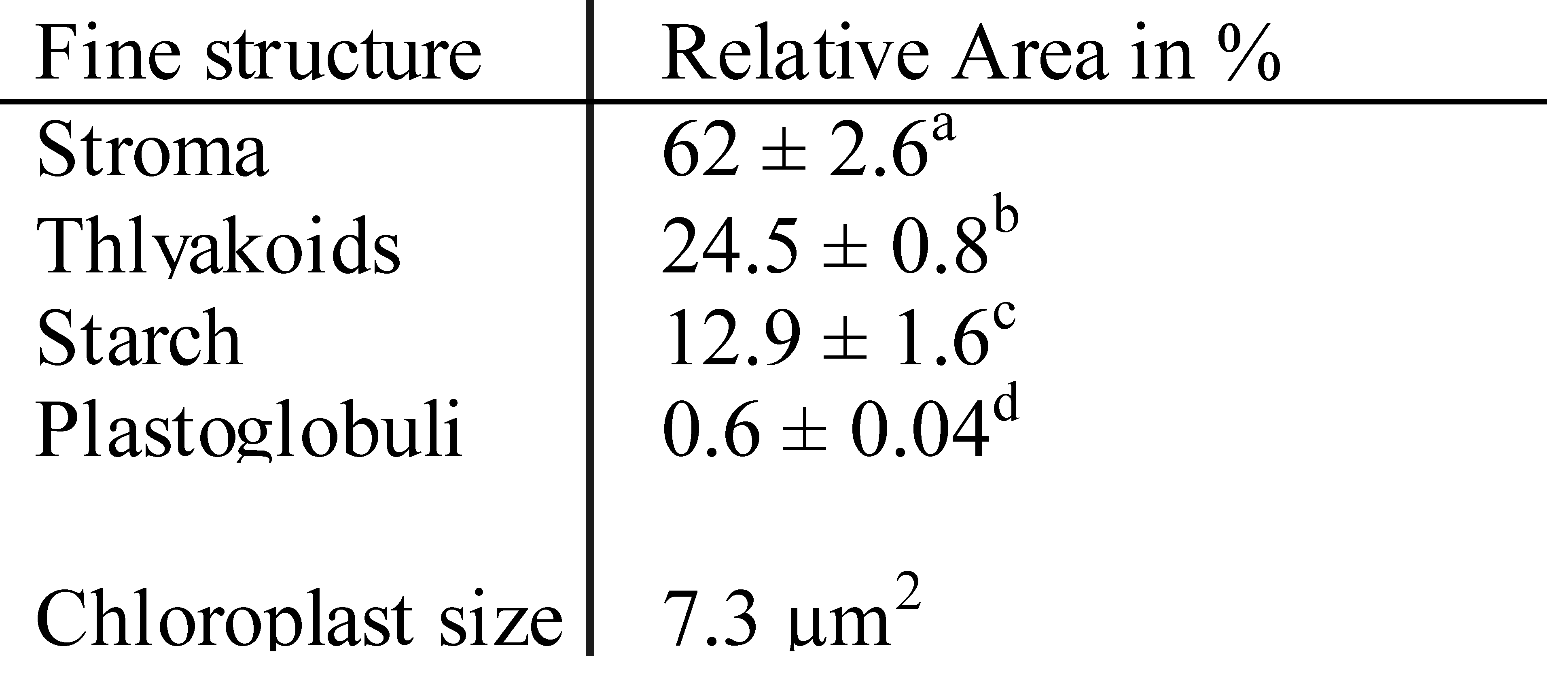

Supplement: Table S2 — Total number and size of chloroplast fine structures in mock inoculated Arabidopsis thaliana Col 0. Values are means with standard errors and document the relative area of internal chloroplast structures and the size of chloroplast in µm2 detected by TEM on a longitudinal ultrathin section within the mesophyll of mock inoculated leaves in Arabidopsis thaliana [L.] Heynh. ecotype Columbia (Col-0). Different lowercase letters indicate significant differences (P<0.05) analyzed with the Kruskal-Wallis test followed by post-hoc comparison according to Conover. N>20 from at least four different samples. (TIF) [file pone.0065811.s007.tif]
